# Comparisons between Direct Anterior Approach and Lateral Approach for Primary Total Hip Arthroplasty in Postoperative Orthopaedic Complications: *A Systematic Review and Meta‐Analysis*

**DOI:** 10.1111/os.13101

**Published:** 2021-08-05

**Authors:** Xiao‐tao Huang, Dong‐guang Liu, Bin Jia, Ying‐xing Xu

**Affiliations:** ^1^ Department of Orthopaedics and Traumatology Cixi Hospital of Traditional Chinese Medicine Ningbo China; ^2^ Department of Orthopaedics and Traumatology Weihai Hospital of Traditional Chinese Medicine Weihai China; ^3^ Department of Joint Surgery The Affiliated Hospital of Qingdao University Qingdao China; ^4^ Qingdao University Qingdao China

**Keywords:** Anterior approach, Complication, Lateral approach, Meta‐analysis, Total hip arthroplasty

## Abstract

The direct anterior approach (DAA) are attracting increasing attention from orthopedic arthroplasty surgeons, due to the less blood loss, mild soft tissue invasion, rapid rehabilitation and shorter length of stay. However, the longer learning curve in DAA can give rise to several complications, such as intraoperative femoral fracture, lateral femoral cutaneous nerve injury, wound‐healing problem, premature revision and so on. This meta‐analysis was performed to compare the rate of postoperative orthopedic complications between the DAA and the lateral approach (LA). All studies involving the comparison of postoperative orthopedic complications after THA between the DAA and LA group were searched in 7 databases prior to October 2020. The odds ratio (*OR*) with the 95% confidence intervals (*CI*) for each outcome was calculated by using the RevMan 5.3. The methodological bias of included studies was evaluated and the potential heterogeneity sources were analyzed. Thirteen comparative studies including a total of 24853 hips (9575 hips in the DAA group and 15278 hips in the LA group) were eligible for this meta‐analysis. There was no significant difference in the rate of surgical site infection [2.59% vs 2.14% (*OR* = 0.98; 95% *CI*: 0.59‐1.61, *P* = 0.93)], heterotopic ossification [12.16% vs 26.47% (*OR* = 0.46; 95% *CI*: 0.20‐1.07, *P* = 0.07)] and reoperation [2.70% and 2.11% respectively (*OR* = 0.93; 95% *CI*: 0.68‐1.26, *P* = 0.64)] between the DAA and LA groups. Although a lower rate in prosthesis malposition [36.19% vs 54.86% (*OR* = 0.50; 95% *CI*: 0.35‐0.73, *P* = 0.0003)], leg length discrepancy [1.87% vs 2.37% (*OR* = 2.35; 95% *CI*: 1.30‐4.25, *P* = 0.005)] and Trendelenburg gait [1.68% vs 4.78% (*OR* = 0.29; 95% *CI*: 0.13‐0.65, *P* = 0.003)] was observed in the DAA group, a higher rate in dislocation [0.77% vs 0.18% (*OR* = 3.73; 95% *CI*: 2.35‐5.94, *P*< 0.00001)], periprosthetic fracture [1.05% vs 0.41% (*OR* = 2.38; 95% *CI*: 1.58‐3.58, *P*< 0.0001)], prosthesis loosening [0.61% vs 0.37% (*OR* = 1.66; 95% *CI*: 1.05‐2.62, *P* = 0.03)] and nerve injury [0.95% vs 0% (*OR* = 7.12; 95% *CI*: 1.66‐30.48, *P* = 0.008)] was found in the DAA group. This meta‐analysis demonstrated several evidences indicating that the DAA exhibited the advantages in the accurate prosthesis placement and less damage of surrounding hip musculature. However, a higher rate in dislocation, periprosthetic fracture, prosthesis loosening and nerve injury in the DAA group should be paid more attention, due to the limited exposure and a longer learning curve, compared to the LA.

## Graphical abstract

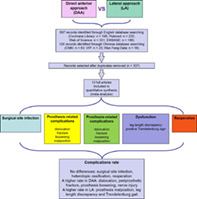

## Introduction

Total hip arthroplasty (THA), an extensive clinical application for patients with painful hip disorders such as osteoarthritis (OA), osteonecrosis of the femoral head (ONFH) and femoral neck fracture, contributes to the excellent results in pain relief and function improvement of the hip joint. The clinical outcomes after THA are associated with various factors: prosthetic design, surgical procedure and perioperative management. Among them, the selection of surgical approaches is a focus of interest in the recent studies^1^, ^2^, ^3^.

The lateral approach (LA), one of the most common surgical approaches used in THA around the world^4^, includes the anterolateral approach (Watson‐Jones)^5^ and the direct lateral approach (Hardinge)^6^. The LA has been developed to maximize surgical visualization, which provides an excellent exposure for the proximal femur and acetabulum. Specifically, the exposure of proximal femur in this approach can be extended as required^1^. Furthermore, this approach is considered to be beneficial to the preservation of posterior soft tissue of the hip joint and avoid the common complications in surgical approaches through the posterior hip joint. Therefore, a lower risk of dislocation has been reported in this approach for THA. The result of a meta‐analysis shows that the dislocation rate in the LA, including anterolateral approach and direct lateral approach, is between 0.43% and 0.70%^7^. Whereas, the lateral approach can lead to the such complications as severe postoperative pain in the early period, heterotopic ossification and damage of superior gluteal nerve, due to the harassment of muscles around hip joint. Moreover, the lateral approach has been reported to extend hospitalization and rehabilitation^1^.

The direct anterior approach (DAA) is a popular surgical approach for THA in recent years and is considered a variant of the Smith‐Peterson anterior approach. The DAA is performed in the interval between the tensor fasciae latae and the sartorius muscles, avoiding splitting the muscle attachments from bone and leading to the less soft tissue disruption around the hip. Therefore, the DAA is advocated by many arthroplasty surgeons based on the following benefits: minimal soft tissue invasion, mild postoperative pain, short hospitalization and rehabilitation^8^. However, it should be noted that the longer learning curve, a major disadvantage of the DAA, can give rise to some complications, including intraoperative femoral fracture or perforation, lateral femoral cutaneous nerve injury, wound‐healing problems and premature revision^9^, ^10^. According to a Bayesian meta‐analysis^11^, the risk incidences of intraoperative trochanter and femoral fractures were 0.8% and 0.5% respectively, and the risk incidence of 2.1% was found for early revisions in DAA for performing THA, leading to DAA becoming a technically demanding surgery approach.

As a result of this, it is still controversial as to the ideal surgical approach of THA. Although the comparison of DAA and LA has been performed by several studies^12^, ^13^, ^14^, ^15^, ^16^, the outcomes are inconsistent, due to the limited samples and methodological differences. To our knowledge, only three meta‐analysis have been published comparing of DAA with LA^17^, ^18^, ^19^, but they all only paid more attention to the differences of Harris hip score, operation time, blood loss, and length of hospital stay; few focused on the postoperative orthopedic complications. As is well known, evaluation of complications is usually the authentic reflection of clinical outcomes and determine the choice of surgical approaches.

Here, we performed a meta‐analysis with the aim to: (i) systematically review applications of the two surgical approaches (DAA and LA) in THA; (ii) conduct a more comprehensive assessment in the following postoperative orthopedic complications of THA: surgical site infection; prosthesis‐related complications (dislocation, fracture, loosening and malposition); surgical trauma‐related complications (nerve injury and heterotopic ossification); dysfunction (leg length discrepancy and Trendelenburg gait); reoperation, and (iii) provide evidence to support the objective chosen for the two surgical approaches (DAA and LA) for THA.

## Materials and Methods

This study was conducted based on the guidelines of the Preferred Reporting Items for Systematic Reviews and Meta‐Analyses statement (PRISMA)^20^.

### Literature Search

A comprehensive search was conducted for the published literatures by four English databases (PubMed, Embase, Web of Science, Cochrane Library) and three Chinese databases (China Knowledge Resource Integrated Database (CNKI), VIP Database, and Wan fang Database) from their inception to October 2020, with the following search terms: (“direct anterior approach” OR “Hueter approach” OR “SmithPetersen approach”) AND (“lateral approach” OR “direct lateral approach” OR “anterolateral approach”) AND (“total hip arthroplasty” OR “total hip replacement” OR “THA” OR “THR”). Moreover, the other relevant studies were collected from the reference lists of retrieved literatures and previous systematic reviews or meta‐analyses.

### Inclusion and Exclusion Criteria

Three investigators (Xiaotao Huang, Dongguang Liu and Bin Jia) independently reviewed the titles and abstracts of articles, and then selected full texts by the following inclusion criteria: (i) study design included randomized controlled trials (RCTs), case–control studies or comparative studies in English or Chinese; (ii) patients suffered from primary THA; (iii) comparisons between DAA and LA were conducted for THA; and (iv) at least one of the following complications were reported: surgical site infection, prosthesis‐related complications(dislocation, fracture, loosening and malposition), surgical trauma‐related complications (nerve injury and heterotopic ossification), dysfunction (leg length discrepancy and positive Trendelenburg sign), reoperation.

Studies were excluded based on the following exclusion criteria: (i) study design included non‐comparative studies, cohort studies, reviews or meta‐analysis, case reports, surgical techniques reports, editorials, letters to editors and animal experiments; (ii) studies involved hemiarthroplasty, computer navigation or robot‐assisted THA; and (iii) studies with incomplete data or incorrect data.

### Data Extraction

Two investigators (Xiaotao Huang and Dongguang Liu) independently extracted data from the included studies according to the following items: (i) first author's surname; (ii) publication year; (iii) studies' methodological features; (iv) characteristics of the cases: sample size, age range, gender ratio; (v) follow‐up time; and (vi) postoperative complications rate (surgical site infection, dislocation, periprosthetic fracture, prosthesis loosening, prosthesis malposition, nerve injury, heterotopic ossification, leg length discrepancy, Trendelenburg gait). If the important data was not available, the listed authors would be contacted to request the original data by email. Disagreements between the two investigators were resolved by discussion and consultation with a senior researcher (Yingxing Xu).

### Assessment of Risk of Bias (ROB) in the Included Studies

Two investigators (Xiaotao Huang and Bin Jia) evaluated the ROB of included studies independently by the Newcastle‐Ottawa Scale (NOS) for non‐randomized studies^21^, and the assessment tool of the Cochrane Collaboration for RCTs^22^. Three sections: selection, comparability and outcome were involved in the NOS. Studies with a score between 0–3 points were considered as low quality, between 4–6 points considered as medium quality, and between 7–9 points considered as high quality. Any discrepancies between the two investigators were resolved by discussion and consultation with a senior researcher (Ying‐xing Xu).

### Statistical Analysis

The Review Manager (RevMan) version 5.3 (Cochrane Collaboration, Oxford, UK) was used to analyze the extracted data. The odds ratio (*OR*) and 95% confidence interval (*CI*) were calculated for the meta‐analysis due to that the postoperative complications rate was binary classification data. The *P* value *<* 0.05 was considered as statistically significant. Meanwhile, the *I* ^2^ value based on standard chi^2^ test was used to assess statistical heterogeneity. When the *P* value > 0.1 and *I* ^*2*^ value <50%, the study was considered as statistically homogeneous, and was assessed by the fixed effects model for meta‐analysis. When the *P* value > 0.1 and *I* ^*2*^ value > 50%, the study was considered as statistically heterogeneous, and then was assessed by the random effects model for meta‐analysis, while analyzing the sources of heterogeneity. The publication bias was evaluated by funnel plot. If an asymmetry was shown in the funnel plot, publication bias existed in the included studies.

## Results

### Search Results

**Fig. 1:**
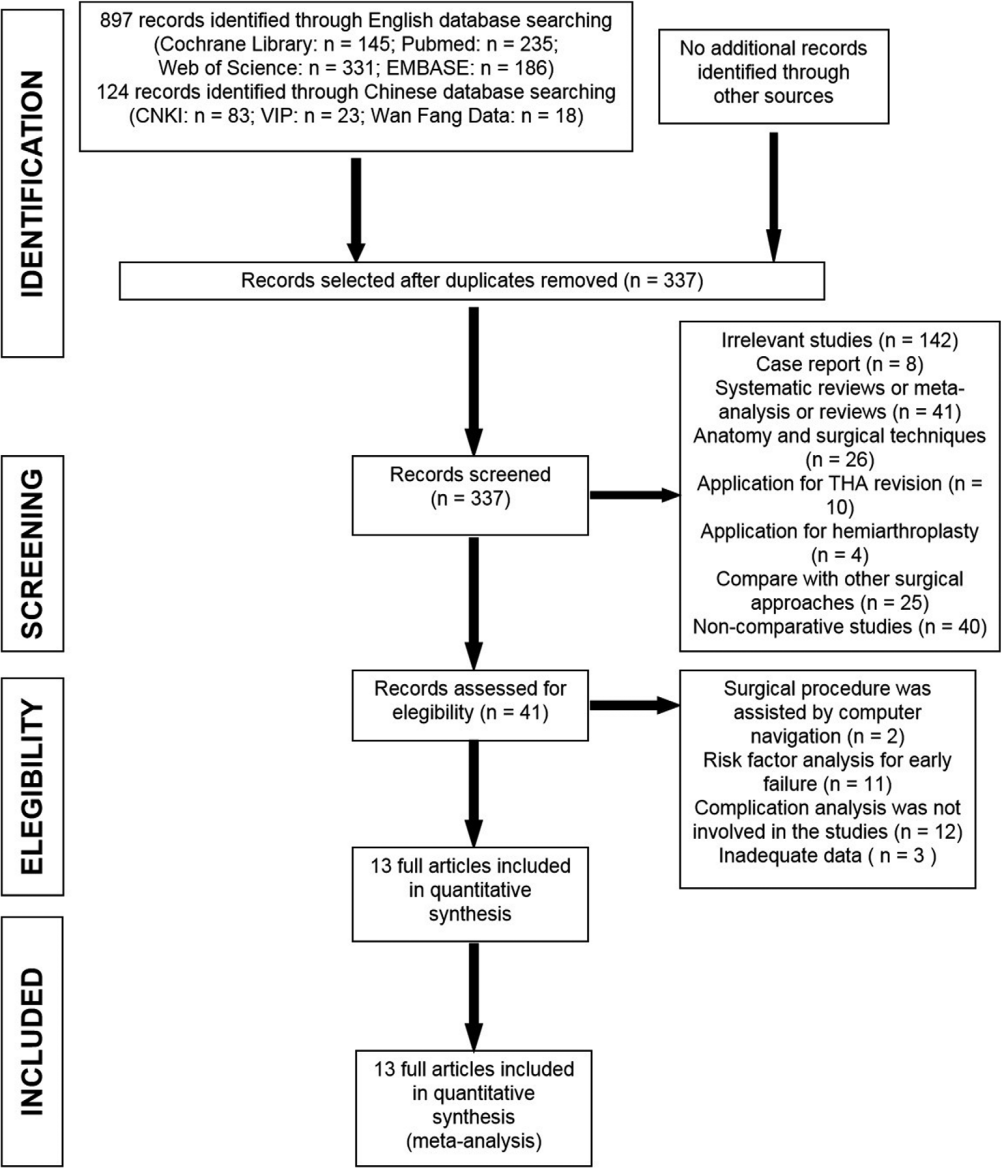
Meta‐analysis flow chart.

A preliminarily review of 1021 articles sourced from the database searches was conducted. After excluding duplicate articles, 337 articles were left. Review of the titles and abstracts according to the inclusion and exclusion criteria resulted in exclusion of a further 296 articles, and full‐text review of the remaining 41 articles resulted in the selection of the final 13 articles published in English. No article was eligible for inclusion from the reference review. Figure 1 shows the search and exclusion process in details.

### Baseline Characteristics of the Included Studies

A total of 13 articles were included in the meta‐analysis, of which, five articles^16^, ^23^, ^24^, ^25^, ^26^ were RCTs and eight articles^15^, ^27^, ^28^, ^29^, ^30^, ^31^, ^32^, ^33^ were case–control studies, including 24,853 hips (9575 hips in the DAA group and 15,278 hips in the LA group). The included articles were published between 2010 and 2019, and the longest period of follow‐up was 3.72 years. Table 1 showed the baseline characteristics of the included studies in details.

**TABLE 1:** Characteristics of included studies in the meta‐analysis

Study (authors, year)	Sample size, *n* (No. of hips)	Study type	Age (years), mean ± SD/range	Gender (*n*),(M/F)	BMI (mean ± SD/range)	Follow‐up, periods	Outcome analyses (complications)
Aggarwal *et al*., 2019 ^31^	DAA(1329), AA(30), DLA(393)	Case–control study	DAA(63), AA(63.9), DLA(61)	DAA(572/757) AA(16/14)DLA(174/219)	DAA(27.7), AA(27.7), DLA(29.9)	3.72 years	Dislocation, infection, periprosthetic fracture, aseptic loosening, leg length discrepancy, nerve damage, prosthesis malposition, HO, reoperation
Brun *et al*., 2019 ^22^	DAA(84), DLA(80)	RCT	DAA (67.2 ± 8.6), DLA (65.6 ± 8.6)	DAA (25/59), DLA (30/50)	DAA (27.7 ± 3.6), DLA (27.6 ± 3.9)	N/A	Prosthesis malposition, leg length discrepancy
Fleischman *et al*., 2019 ^28^	DAA(5465), DLA(8561)	Case–control study	DAA(62.7 ± 11.2), DLA(63.4 ± 11.7)	DAA (2814/2651), DLA (4375/4186)	DAA(28.4 ± 5.1), DLA(29.9 ± 5.5)	90 days, 1 year, 2 years	Dislocation/instability, periprosthetic fracture, aseptic loosening, revision
Hart *et al*., 2019 ^32^	DAA(293), LA(565)	Case–control study	DA (64.3 ± 10.9), LA (67.9 ± 12.2)	DAA (124/169), LA (269/296)	DA (29.9 ± 5.3), DLA (30.6 ± 7.4)	30 days	Infection, leg length discrepancy, Trendelenburg gait, reoperation
Mjaaland *et al*., 2019 ^23^	DAA(84), DLA(80)	RCT	DAA (67 ± 9), DLA (66 ± 9)	DAA (25/59), DLA (30/50)	DAA (28 ± 4), DLA (28 ± 4)	3, 6, 12, and 24 months	Dislocation, infection, nerve injury, periprosthetic fracture, Trendelenburg gait, reoperation
Takada *et al*., 2018 ^25^	DAA(30) AA(30)	RCT	62.6 ± 10.4	DAA(4/26) AA(4/26)	24.4 ± 4.4	1 year	Dislocation, infection, periprosthetic fracture, nerve injury, leg length discrepancy, Trendelenburggait
Zomar *et al*., 2018 ^21^	DAA(36), DLA(42)	RCT	DAA(60.78 ± 9.26), DLA(59.54 ± 8.4)	DAA(21/15), DLA(20/22)	DAA(28.38 ± 4.51), DLA(30.89 ± 5.43)	2, 6, 12 weeks	Periprosthetic fracture
Hürlimann *et al*., 2017 ^30^	DAA(39), AA(67)	Case–control study	DAA(39), AA(38.5)	DAA(22/17), AA(31/36)	N/A	1 year	Dislocation, infection, periprosthetic fracture, loosening, leg length discrepancy, Trendelenburg gait, HO, revision
Chen *et al*., 2016 ^27^	DAA(186), DLA(186)	Case–control study	DAA(67.7 ± 9.8), DLA(68 ± 10.8)	DAA(97/89), DLA(92/94)	DAA(30.2 ± 5.1), DLA(30.1 ± 5.2)	4 weeks, 6 months, 1 year	Dislocation, infection, loosen, prosthesis malposition
Gromov *et al*., 2016 ^26^	DAA(93), AA(166), DLA(101)	Case–control study	62.8(25–75)	441/395	28.5(18.5–51.1)	N/A	Prosthesis malposition
Sheth *et al*., 2015^15^	DAA(1851), AA(4226), DLA(667)	Case–control study	DAA(65 ± 11), AA(67 ± 11), DLA(65 ± 11)	DAA(736/1112), AA(1788/2435), DLA(281/385)	DAA(28 ± 5), AA(29 ± 6), DLA(30 ± 6)	3 years	Dislocation, revision
Pogliacomi *et al*., 2012 ^29^	DAA(35), DLA(35)	Case–control study	DAA(64.6, 46–79), DLA(64.5, 48–80)	DAA(19/16), DLA(18/17)	DAA(26.6 ± 1.76), DLA(26.5 ± 1.93)	1 year	Dislocation, infection, nerve injury, prosthesis malposition, HO
Restrepo *et al*., 2010 ^24^	DAA(50), DLA(49)	RCT	DAA(62.02, 35.0–84.5), DLA(59.91, 40.1–76.1)	DAA(17/33), DLA(22/27)	DAA(25.18, 18.8–29.9), DLA(25.17, 19.2–29.1)	2 years	Periprosthetic fracture, nerve injury, Trendelenburg gait

AA, anterolateral approach; BMI, body mass index; DAA, direct anterior approach; DLA, direct lateral approach; HO, heterotopic ossification; LA, lateral approach; NA, not available; RCT, randomized controlled trial; SD, standard deviation.

### ROB in the Included Studies

**Fig. 2:**
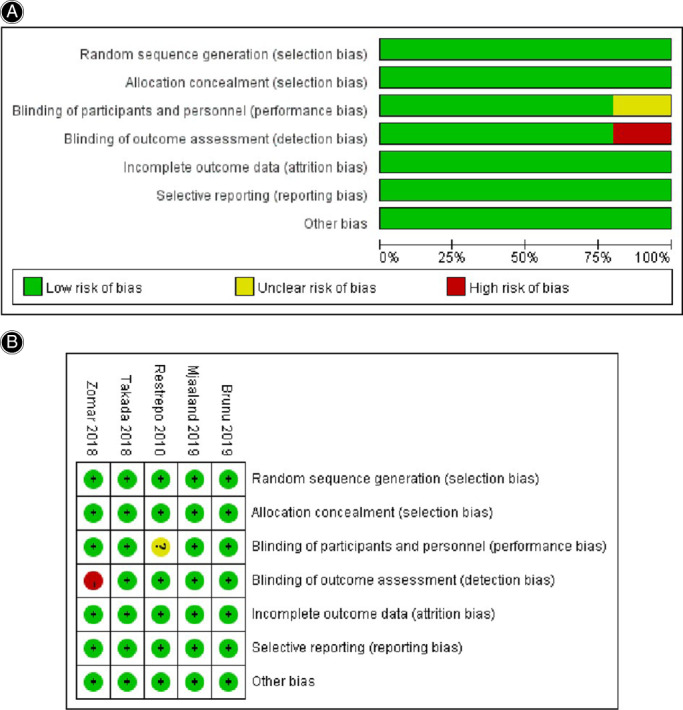
The methodological quality assessment for RCTs. (A) Risk‐of‐bias graph for included studies; (B) Risk‐of‐bias summary for included studies. +: no bias; −, bias;?: bias unknown.

The assessment tool of Cochrane Collaboration and NOS was utilized to evaluate the methodological qualities of RCT and case–control studies, respectively. The risk‐of‐bias summary and graph in Fig. 2 shows that the five RCT studies^16^, ^23^, ^24^, ^25^, ^26^ were of high quality, of these, two were therapeutic studies at evidence level 1 reported in the publication. In addition, NOS scores for eight case–control studies^15^, ^27^, ^28^, ^29^, ^30^, ^31^, ^32^, ^33^ were at least seven points, suggesting that the methodologic quality of these studies was relatively stable (Table 2).

**TABLE 2:** Quality assessment of case–control studies

Study	Is the case definition adequate?	Representativeness of the cases	Selection of Controls	Definition of Controls	Comparability of cases and controls on the basis of the design or analysis	Ascertainment of exposure	Same method of ascertainment for cases and controls	Non‐Response rate	Total
Aggarwal *et al*., 2019 ^31^	1	1	1	1	1	1	1	1	8
Fleischman *et al*., 2019 ^28^	1	1	1	1	2	1	1	1	9
Hart *et al*., 2019 ^32^	1	1	1	1	1	1	1	1	8
Hürlimann *et al*., 2017 ^30^	1	1	1	1	1	1	1	1	8
Chen *et al*., 2016 ^27^	1	1	1	1	1	1	1	1	8
Gromov *et al*., 2016 ^26^	1	1	1	1	1	1	1	0	7
Sheth *et al*., 2015 ^15^	1	1	1	1	1	1	1	1	8
Pogliacomi *et al*., 2012 ^29^	1	1	1	1	2	1	1	1	9

### Surgical Technique

**Fig. 3:**
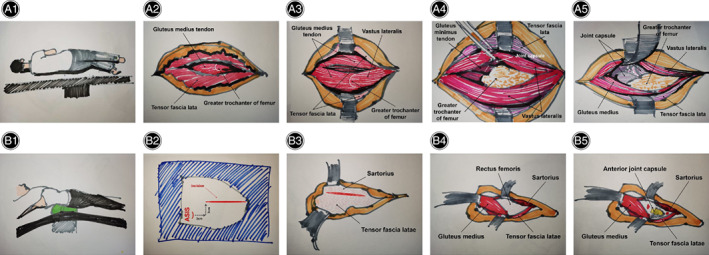
Technique schematic illustration for the LA and DAA. (A) The technique diagrams for the LA shows: (1) lateral recumbent position; (2) incision of tensor fasciae lata and exposure for gluteus medialis tendon; (3) incision of gluteus medius and vastus lateralis; (4) incision of gluteus minimus tendon and exposure for joint capsule; (5) incision of and exposure for femoral neck. (B) The technique diagrams for the DAA shows: (1) supine position; (2) skin incision; (3) exposure for tensor fascia lata; (4) exposure for rectus femoris and gluteus medius; (5) exposure for anterior articular capsule. DAA, direct anterior approach; LA, lateral approach.

The lateral approach was performed with the following procedures^1^: the procedure began by positioning the patient in the lateral decubitus position (Fig. 3A1). A longitudinal incision was performed extending about 5 cm proximal and 8 cm distal to the tip of the greater trochanter. Then, the tensor fascia lata was incised to expose the gluteus medius tendon in line with the skin incision (Fig. 3A2). The gluteus medius was split from the tip of the greater trochanter, and the lateral vastus lateralis was extended about 2 cm to the distal end (Fig. 3A3). Subsequently, the gluteus minimus tendon was split to expose the anterior joint capsule (Fig. 3A4). Finally, the femoral neck was exposed after the joint capsule was incised (Fig. 3A5).

DAA was performed with the following procedures^1^: The surgery began by positioning the patient in the supine position (Fig. 3B1). The surgical incision was designed to be 3 cm distal and anterior to the anterior superior iliac spine (ASIS) (Fig. 3B2). The superficial fascia of tensor fascia latae was identified and incised (Fig. 3B3). Next, the tensor fascia lata and sartorius were retracted after identification for the interval between them, and then rectus femoris and gluteus medius were exposed (Fig. 3B4). The hip retractor displaced the rectus femoris medially and the gluteus medius laterally to expose the anterior joint capsule of the hip (Fig. 3B5). Finally, the femoral neck was exposed after the joint capsule was incised.

### Surgical Site Infection

**Fig. 4:**
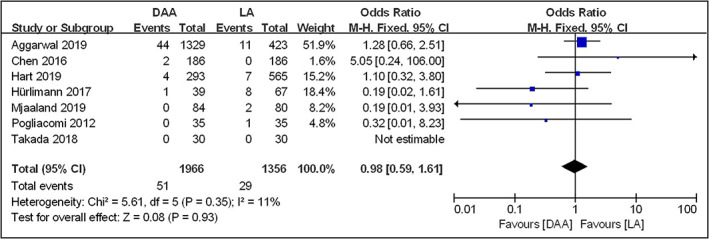
Forestplot for comparison of surgical site infection rate between the DAA and LA groups. DAA, direct anterior approach; LA, lateral approach; Fixed, fixed‐effects modeling; M‐H, Mantel‐Haenszel; CI, confidence intervals; df, degrees of freedom.

Although a total of seven articles^16^, ^26^, ^27^, ^28^, ^29^, ^30^, ^31^ were involved in surgical site infection including superficial or deep infection, six studies^16^, ^27^, ^28^, ^29^, ^30^, ^31^ were included in the meta‐analysis to evaluate the surgical site infection rate, except for the study of Takada *et al*.^26^ due to that no cases of surgical site infection were reported in both DAA and LA groups. Among of them, two studies^28^, ^29^ reported superficial infection, one study^27^ reported deep infection such as periprosthetic joint infection (PJI), and three studies^16^, ^30^, ^31^ involved both superficial and deep infection. Fixed effects model was adopted in the meta‐analysis because of small heterogeneity among the included studies (*I* ^2^  = 11%, *P* = 0.35). The results showed that the surgical site infection rate in the DAA group (1966 hips) and the LA group (1356 hips) was 2.59% and 2.14% respectively (*OR* = 0.98; 95% *CI* 0.59–1.61, *P* = 0.93), indicating that there was no difference in surgical site infection rate between the two groups (Fig. 4).

### Prosthesis‐related Complications

#### Dislocation

**Fig. 5:**
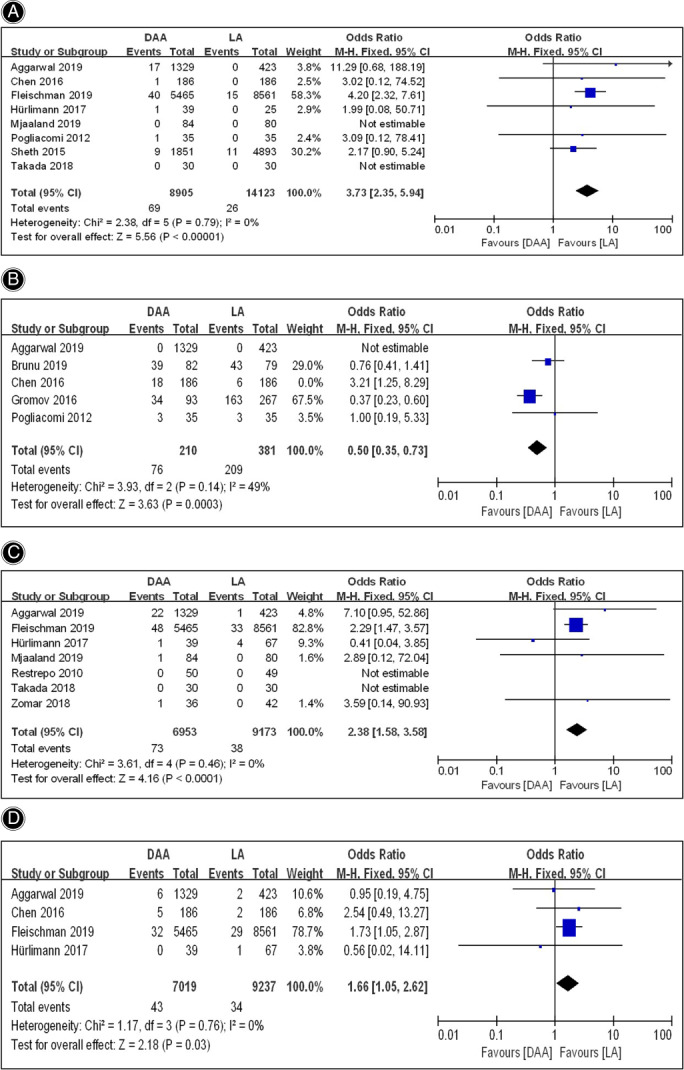
Forestplot for comparison of prosthesis‐related complications rate between the DAA and LA groups. (A) Comparison of dislocation rate between the two groups; (B) Comparison of prosthesis malposition rate between the two groups; (C) Comparison of periprosthetic fracture rate between the two groups; (D) Comparison of prosthesis loosening rate between the two groups. DAA, direct anterior approach; LA, lateral approach; Fixed, fixed‐effects modeling; M‐H, Mantel–Haenszel; CI, confidence intervals; df, degrees of freedom.

As shown in Fig. 5A, a total of eight studies^15^, ^16^, ^26^, ^27^, ^28^, ^29^, ^30^, ^32^ including mentioned dislocation, which was the most common complication of prosthesis. Considering that no dislocation cases occurred in both DAA and LA group in the study of Mjaaland *et al*.^16^ and Takada *et al*.^26^, six studies^15^, ^27^, ^28^, ^29^, ^30^, ^32^ involving 23,028 hips in all were included in the meta‐analysis to assess the postoperative dislocation rate. The meta‐analysis for fixed effects model (*I* ^2^ = 0%, *P* = 0.79) showed that the postoperative dislocation rate in DAA group and LA group was 0.77% and 0.18% respectively (*OR* = 3.73; 95% *CI* 2.35–5.94, *P* < 0.00001), suggesting that the postoperative dislocation rate in DAA group was significantly higher than that in LA group.

#### Prosthesis Malposition

Although the comparison of prosthesis malposition rate between the DAA and LA group was reported in five articles^24^, ^27^, ^28^, ^30^, ^33^, of these, onlyfour were included in the meta‐analysis as the the study of Aggarwal *et al*.,^30^ was excluded due to tthere were no cases of prosthesis malposition in the two groups. Fixed effects model meta‐analysis showed the significant heterogeneity (*I* ^2^ = 82%, *P* = 0.0008). Sensitivity analysis showed that the heterogeneity was reduced significantly (*I* ^2^ = 49%, *P* = 0.14) after excluding the result of Chen *et al*.,^27^ indicating that this study was the primary source of heterogeneity. The final meta‐analysis, including 210 hips in DAA group and 381 hips in LA group, suggested that the prosthesis malposition rate in DAA group (36.19%) was significantly lower than that in LA group (54.86%) (*OR* = 0.50; 95% *CI* 0.35–0.73, *P* = 0.0003) (Fig. 5B).

#### Periprosthetic Fracture

A total of seven studies^16^, ^23^, ^25^, ^26^, ^29^, ^30^, ^32^ reported the comparison of periprosthetic fracture rate between the DAA and LA group, but the studies of Restrepo *et al*.^25^ and Takada *et al*.^26^ were not estimable because no cases of periprosthetic fracture occurred in the two groups. Therefore, five studies^16^, ^23^, ^29^, ^30^, ^32^ were included in the final meta‐analysis. The meta‐analysis for fixed effects model (*I* ^2^ = 0%, *P* = 0.46) showed that the periprosthetic fracture rate in the DAA group (6953 hips) and the LA group (9173 hips) was 1.05% and 0.41% respectively (*OR* = 2.38; 95% *CI* 1.58–3.58, *P* < 0.0001), indicating that the periprosthetic fracture rate in the DAA group was higher than that in the LA group (Fig. 5C).

#### Prosthesis Loosening

The comparison of prosthesis loosening rate between the DAA and LA groups was recorded in four articles^27^, ^29^, ^30^, ^32^. The meta‐analysis for fixed effects model (*I* ^2^ = 0%, *P* = 0.76) showed that the prosthesis loosening rate in the DAA group (7019 hips) and the LA group (9237 hips) was 0.61% and 0.37% respectively (*OR* = 1.66; 95% *CI* 1.05–2.62, *P* = 0.03), indicating that the prosthesis loosening rate in the DAA group was higher than that in the LA group (Fig. 5D).

### Surgical Trauma‐related Complications

#### Nerve Injury

**Fig. 6:**
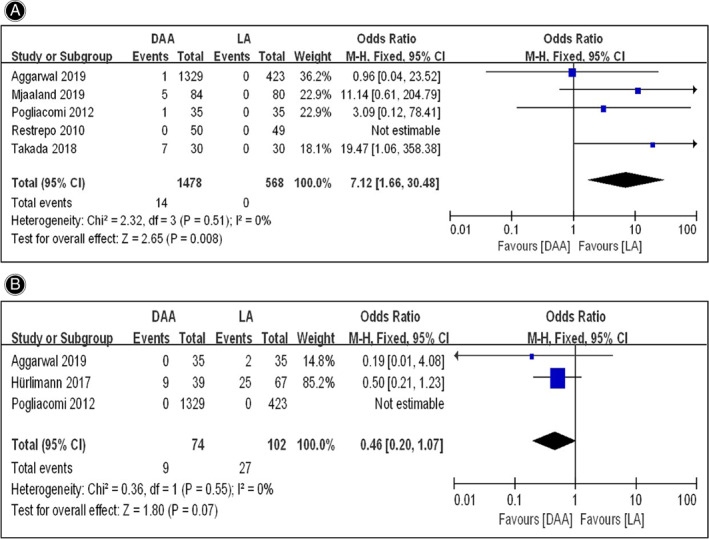
Forestplot for comparison of surgical trauma‐related complications rate between the DAA and LA groups. (A) Comparison of nerve injury rate between the two groups; (B) Comparison of heterotopic ossification rate between the two groups. DAA, direct anterior approach; LA, lateral approach; Fixed, fixed‐effects modeling; M‐H, Mantel‐Haenszel; CI, confidence intervals; bdf, degrees of freedom.

A total of five studies^16^, ^25^, ^26^, ^28^, ^30^ reported the comparison of nerve injury rate between the DAA and LA groups, but the study of Restrepo *et al*.^25^ was not estimable because no cases of nerve injury occurred in the two groups. Therefore, four studies^16^, ^26^, ^28^, ^30^ were included in the final analysis. The meta‐analysis for the fixed effects model (*I* ^2^ = 0%, *P* = 0.51) showed that the nerve injury rate in the DAA group (1478 hips) and the LA group (468 hips) was 0.95% and 0% respectively (*OR* = 7.12; 95% *CI* 1.66–30.48, *P* = 0.008), indicating that the nerve injury rate in the DAA group was higher than that in the LA group (Fig. 6A).

#### Heterotopic Ossification

Although the comparison of heterotopic ossification rate between the DAA and LA groups was reported in three studies^28^, ^29^, ^30^, the study of Pogliacomi *et al*.^28^ was not estimable because no cases of heterotopic ossification occurred in the two groups. Therefore, only two studies^29^, ^30^ were included in the final analysis. The meta‐analysis for fixed effects model (*I* ^2^ = 0%, *P* = 0.55) showed that the heterotopic ossification rate in the DAA group (74 hips) and the LA group (102 hips) was 12.16% and 26.47% respectively (*OR* = 0.46; 95% *CI* 0.20–1.07). However, there was no statistical difference in heterotopic ossification rate between the two groups (*P* = 0.07) owing to the limited sample size (Fig. 6B). In view of this, the pooling result should be taken with caution.

### Dysfunction

#### Leg Length Discrepancy

**Fig. 7:**
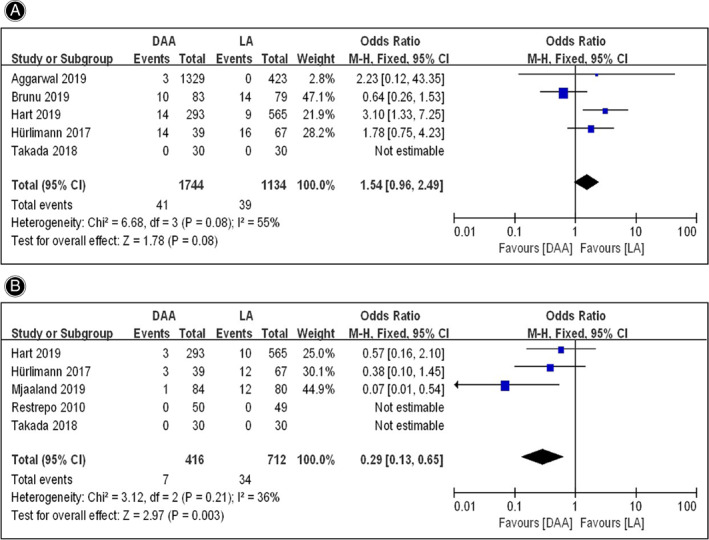
Forestplot for comparison of dysfunction rate between the DAA and LA groups. (A) Comparison of leg length discrepancy rate between the two groups; (B) Comparison of Trendelenburg gait rate between the two groups. DAA, direct anterior approach; LA, lateral approach; Fixed, fixed‐effects modeling; M‐H, Mantel‐Haenszel; *CI*, confidence intervals; *df*, degrees of freedom.

Because no cases of leg length discrepancy occurred in the two groups in the study of Takada *et al*.^26^, a total of four articles^24^, ^29^, ^30^, ^31^ were included in the meta‐analysis. The fixed effects model meta‐analysis showed significant heterogeneity (*I* ^2^ = 55%, *P* = 0.08). Sensitivity analysis showed that the heterogeneity was reduced significantly (*I* ^2^ = 0%, *P* = 0.67) after excluding the results of Brun *et al*.^24^, indicating that this study was the primary source of heterogeneity. The final meta‐analysis, including 1661 hips in the DAA group and 1055 hips in the LA group, suggested that the leg length discrepancy rate in the DAA group (1.87%) was significantly lower than that in the LA group (2.37%) (*OR* = 2.35; 95% *CI* 1.30–4.25, *P* = 0.005) (Fig. 7A).

#### Trendelenburg Gait

A total of three articles^16^, ^29^, ^31^ were included in the meta‐analysis, because no cases of Trendelenburg gait occurred in the two groups in the studies of Restrepo *et al*.^25^ and Takada *et al*.^26^. The meta‐analysis for fixed effects model (*I* ^2^ = 36%, *P* = 0.21) showed that the Trendelenburg gait rate in the DAA group (416 hips) and the LA group (712 hips) was 1.68% and 4.78% respectively (*OR* = 0.29; 95% *CI* 0.13–0.65, *P* = 0.003), indicating that the Trendelenburg gait rate in the DAA group was lower than that in the LA group (Fig. 7B).

#### Reoperation

**Fig. 8:**
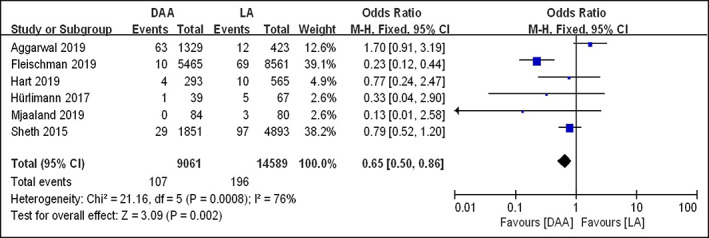
Forestplot for comparison of reoperation rate between the DAA and LA groups. DAA, direct anterior approach; LA, lateral approach; Fixed, fixed‐effects modeling; M‐H, Mantel–Haenszel; *CI*, confidence intervals; *df*, degrees of freedom.

A total of six studies^15^, ^16^, ^29^, ^30^, ^31^, ^32^ reported the comparison of reoperation rate between the DAA and LA group, all of them were included in the final meta‐analysis. Fixed effects model meta‐analysis showed the significant heterogeneity (*I* ^2^ = 76%, *P* = 0.0008). Sensitivity analysis showed that the heterogeneity was reduced significantly (*I* ^2^ = 42%, *P* = 0.14) after excluding the result of Fleischman *et al*.^32^, indicating that this study was the primary source of heterogeneity. The final meta‐analysis suggested that the reoperation rate in DAA group (3596 hips) and LA group (6028 hips) was 2.70% and 2.11% respectively (*OR* = 0.93; 95% *CI* 0.68–1.26, *P* = 0.64), indicating that there was no difference in reoperation rate between the two groups (Fig. 8).

## Discussion

Distinguished from the meta‐analysis published in the past^17^, ^18^, ^19^, the present study paid more attention to the comparison of postoperative complications between the DAA and LA for THA, such as surgical site infection, prosthesis‐related complications (dislocation, fracture, loosening and malposition), surgical trauma‐related complications (nerve injury and heterotopic ossification), dysfunction (leg length discrepancy and Trendelenburg gait) and reoperation.

In terms of surgical site infection, it qas established that the postoperative infection of THA qas closely related to wound size, operation time, blood loss and intraoperative fluoroscopy. Several previous studies^34^, ^35^, ^36^ have suggested that a higher rate of wound complications and infection was found in the DAA compared with other approaches and considered that the obese patients with BMI ≥35 kg/m^2^ were important risk factors of surgical site infection after THA by anterior approach. However, in the present meta‐analysis, no significant difference was found in the rate of surgical site infection between the DAA and LA groups, which may be related to the BMI of less than 35 kg/m^2^ in included patients.

Dislocation, periprosthetic fracture, prosthesis loosening and malposition were involved in the analysis for prosthesis‐related complications. Of these, dislocation or instability is the most common complication and was often considered to be closely related to surgical approach. The previous studies generally agreed that DAA could reduce the incidence of hip dislocation after THA compared with posterior approaches (PA), due to the minimal disruption of the posterior capsule and short external rotators^37^, ^38^. However, a lower risk of dislocation or instability was found in laterally based approaches including anterolateral and direct lateral approach^15^, ^30^, ^39^. As previously reported, our meta‐analysis demonstrated that the postoperative dislocation rate in the DAA group was significantly higher than that in the LA group. In addition, prosthesis malposition is another important cause of postoperative dislocation. In this meta‐analysis, we evaluated such prosthesis malposition as improper cup anteversion, abduction and stem alignment between the DAA and LA groups and found that the prosthesis malposition rate in the DAA group was significantly lower than that in the LA group, which showed the superiority of DAA on the correct placement of the prosthesis. As result of this, we speculated that compared with the LA, the higher risk of postoperative dislocation in DAA may be related not to the prosthesis malposition but the release of the tendon and capsule around the hip.

Furthermore, a longer learning curve was often considered as the most significant shortcoming of DAA for THA. During the learning curve, a higher rate of femoral failure, including periprosthetic femoral fracture and aseptic loosening, was reported in the DAA in previous studies^40^, ^41^, ^42^, ^43^ due to the difficulty with the exposure for preparing the femur and implanting the prosthesis. By contrast, LA could provide better acetabular and femoral exposure^1^, ^44^. As previously reported, our meta‐analysis suggested that both the periprosthetic fracture and prosthesis loosening rate in the DAA group was significantly higher than that in the LA group.

In the aspect of nerve injury, the traditional view is that more attention should be paid to the lateral femoral cutaneous nerve (LFCN) in DAA^45^, ^46^ and the superior gluteal nerve (SGN) in LA^47^, ^48^ due to the anatomical nerve distribution. However, an anatomical study demonstrated that DAA could also increase the potential risk of SGN injury because of the coagulation of ascending branch of lateral circumflex femoral artery and the placement of hooks^49^. In addition, peroneal nerve and femoral nerve damage were also observed in the patients after THA by using DAA^50^, ^51^, although the cause of that was unclear. In this meta‐analysis, we found that a higher total rate of nerve injury was observed in the DAA group compared with the LA group. On the other hand, the previous study confirmed the relevance between the rate of heterotopic ossification after THA and the surgical approach used, and found that the Watson‐Jones approach, also called direct lateral approach (DLA), showed a significantly higher heterotopic ossification rate due to the extensive traumatic dissection^52^. Instead, DAA showed the mild muscle injury, less bleeding, and faster rehabilitation time^1^, ^9^. Therefore, it is reasonable to presume that DAA has a lower heterotopic ossification rate than LA used for THA. However, the present meta‐analysis demonstrated that there was no statistical difference in heterotopic ossification rate between the two groups. It could be speculated that the reason for this result may be related to the limited study samples.

With regards to the complications for dysfunction, leg length discrepancy and Trendelenburg gait, were included in this meta‐analysis. Leg length discrepancy has been confirmed to correlate with the pain, excessive prosthesis wear, loosening and instability^53^. However, it is still controversial whether there is a difference in leg length discrepancy between patients after THA using the DAA and LA approaches. Although the study of Debi *et al*.^54^ showed a good outcome in LLD values in patients who underwent THA by the DAA compared to the anterolateral approach (ALA), most authors stated that both DAA and ALA showed similar results in postoperative leg length control^55^, ^56^. In our meta‐analysis, we found that a relatively low rate of leg length discrepancy was observed in the DAA group compared to the LA group. We speculated that supine position used in either DAA and ALA was more conducive to the accurate prosthesis placement and limb length control, but the DLA under lateral decubitus position was included in this meta‐analysis leading to the relatively high rate of leg length discrepancy in the LA group. In addition, Trendelenburg gait was another important indictor to evaluate the postoperative function of patients after THA^57^. To our knowledge, the LA approach could impact gait mechanics of the patients with THA leading to a Trendelenburg gait, because of the surgical release and disruption of the abductor musculature^58^, ^59^. By contrast, DAA was performed through the space between sartorius and tensor fascia latae avoiding the damage of surrounding hip musculature^9^, ^11^. As expected, a lower rate of Trendelenburg gait was found in the DAA group compared to the LA group in this meta‐analysis, although it was reported that abductor muscle damage was also observed in cadavers that underwent THA using DAA due to the release of piriformis and tensor fascia lata for more sufficient exposure^60^.

Furthermore, reoperation was considered as an undesirable and serious postoperative issue in THA, involving debridement and prosthesis revision for various infectious or non‐infectious factors. In spite of previous studies, there was no difference in the risk of reoperation for periprosthetic fractures and aseptic loosening under different surgical approaches^61^, some authors have noted a higher reoperation rate in the DAA group due to the wound drainage and infection^34^, ^35^, ^36^, and several studies have reported the reoperation cases for gluteal insufficiency after THA by using the DLA^16^. In this meta‐analysis, our result showed that there was no difference in reoperation rate between the DAA and LA groups.

In addition, several limitations in this meta‐analysis still need to be considered. First, we were unable to control heterogeneous factors such as surgical knowledge of approaches, patient characteristics and perioperative management. Also, among the 13 included articles, only five were RCTs, indicating that the level of evidence provided was limited. Moreover, the funnel plot used to evaluate the publication bias was not performed in this meta‐analysis, due to a small number of included studies for each complication. Therefore, larger multi‐centre RCTs need to be performed to update the results of our meta‐analysis.

### Conclusion

Collectively, based on the results of our meta‐analysis, although there was no difference in the rate of surgical site infection, heterotopic ossification and reoperation between the DAA and LA groups, a lower rate in prosthesis malposition, leg length discrepancy and Trendelenburg gait were observed in the DAA group, exhibiting the advantages of DAA in the accurate prosthesis placement and less damage of surrounding hip musculature. However, a higher rate in dislocation, periprosthetic fracture, prosthesis loosening and nerve injury was also found in the DAA group, suggesting that the exposure provided by DAA was relatively limited and a longer learning curve for DAA needs to be overcome. Keeping this in mind, the key to reducing the complications of THA depends on familiarity of the surgical approach.
